# p53 and MYC-regulated squalene epoxidase as Achilles heel in colorectal cancer

**DOI:** 10.7150/ijbs.89237

**Published:** 2023-09-04

**Authors:** Martin Fischer

**Affiliations:** Computational Biology Group, Leibniz Institute on Aging - Fritz Lipmann Institute (FLI), Beutenbergstraße 11, 07745 Jena, Germany

**Keywords:** tumor suppressor p53, transcription factor MYC, SQLE, cholesterol synthesis, Terbinafine, colorectal cancer

## Abstract

The transcription factors p53 and MYC are often considered non-druggable targets, but their dysregulation can generate new dependencies and treatment opportunities in cancer cells. The p53 and MYC-regulated squalene epoxidase (SQLE) has been identified as a potential Achilles heel in colorectal cancer. This is of great interest because the FDA-approved anti-fungal SQLE inhibitor Terbinafine could be repurposed to treat colorectal cancer patients.

## Introduction

The transcription factor and tumor suppressor p53 is inactivated in the majority of human cancers. While about half of all cancers harbor p53 gene mutations that generate dysfunctional p53 mutants, wild-type p53 is often inactivated by other means, such as viral oncoproteins or MDM2 amplification. Consequently, p53's ability to *trans*-activate its target genes, which are central to its tumor suppressor function, is perturbed in cancer cells 1. In contrast to the tumor suppressor p53, the transcription factor MYC (also known as c-Myc) induces cell growth and proliferation and is among the most commonly activated oncoproteins in human cancer 2. Colorectal cancer, for example, often displays both mutated p53 and activated MYC 3.

The transcription factors p53 and MYC are both difficult to target therapeutically, but their dysregulation in cancer cells can generate new dependencies that provide treatment opportunities. In a recent study, the Hermeking group identified the squalene epoxidase (SQLE), a rate-limiting enzyme in the cholesterol biosynthesis, as a candidate target in colorectal cancer that harbors p53 mutation and elevated MYC activity 4. It is the latest in a series of studies highlighting SQLE as a pharmaceutically exploitable target in colorectal cancer 5-7.

By combining meta-analyses on p53 and MYC-dependent gene expression, Du *et al*. identified *SQLE* mRNA to be down-regulated by p53 and up-regulated by MYC 4. Confirming earlier studies 5,6, they found *SQLE* to be consistently higher expressed in colorectal cancer compared with normal tissue and to correlate with poor patient survival, and they corroborated that SQLE inhibition, such as through the SQLE inhibitor Terbinafine, decreased the viability of colorectal cancer cells. Importantly, Du *et al*. discovered that the sensitivity of colorectal cancer cells to SQLE inhibition significantly correlated with the cells' p53 status and the dependency on MYC 4.

**Figure 1:**
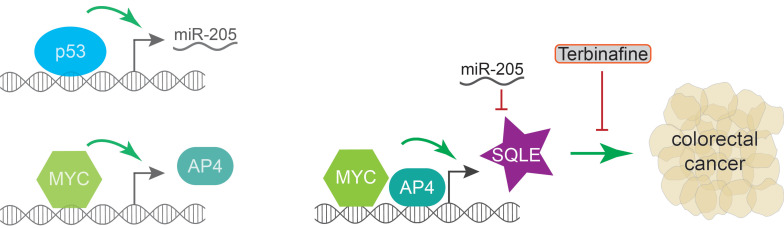
** p53 and MYC-dependent *SQLE* regulation.** *SQLE* mRNA expression is down-regulated by p53 and up-regulated by MYC. p53 employs its direct target *miR-205* to repress *SQLE* expression. MYC directly binds to and activates the *SQLE* promoter. In addition, MYC induces its target AP4, a transcription factor that also binds to the *SQLE* promoter and synergizes with MYC. SQLE promotes colorectal cancer growth and its inhibition, such as through the FDA-approved SQLE inhibitor Terbinafine, decreases viability of colorectal cancer cells. Based on findings from 4.

In their study, Du *et al*. revealed how p53 and MYC regulate the expression of *SQLE* (**Figure 1**). Given that p53 functions as a *trans*-activator that is known to employ downstream factors to mediate the repression of gene expression 1, the authors investigated multiple candidate pathways through which p53 may reduce *SQLE* levels. Ultimately, they uncovered that p53 employs the micro RNA (miR) 205, a miR that is directly *trans*-activated by p53, to down-regulate SQLE expression. Mechanistically, miR-205 binds the 3'UTR of *SQLE* mRNA to promote its degradation 4. Taking the activation of *SQLE* by MYC into account, Du *et al*. found that MYC uses a two-pronged approach to induce SQLE expression. One, MYC directly binds to the *SQLE* promoter to up-regulate its expression, and, two, MYC employs its target AP4, another *trans*-activating transcription factor, which also binds to the *SQLE* promoter and synergizes with MYC in activating *SQLE* expression. Importantly, the induction of SQLE by ectopic MYC or AP4 was paralleled by an increase in cholesterol levels. Understanding the regulation of SQLE by p53 and MYC offers an explanation for the p53 and MYC-dependent vulnerability of colorectal cancer to SQLE inhibition 4.

SQLE is a particularly exciting candidate target for the treatment of colorectal cancer because a well-established SQLE inhibitor, Terbinafine, is FDA-approved for anti-fungal treatment 8 and has been shown to efficiently block SQLE activity and cell growth in multiple models of colorectal cancer, including organoids and xenograft mice 4-7. Moreover, Terbinafine has been shown to have potential synergistic effects with chemotherapeutic agents in inhibiting colorectal cancer growth 6.

In contrast to the overall oncogenic properties of SQLE, Jun *et al*. reported that a reduction of SQLE accelerated the progression and metastasis of aggressive stage IV colorectal cancer. However, this effect was p53-dependent, as experimental metastasis was not observed when p53-deficient cells were used 9. In line with these data, the findings by Du *et al*. suggest that the p53 mutation status and elevated MYC/AP4 levels may be important indications for the treatment of colorectal cancer with Terbinafine 4.

Taken together, the SQLE inhibitor Terbinafine appears to be a promising drug to be repurposed for the treatment of colorectal tumors that carry a p53 mutation and display elevated expression of MYC.
